# *N*-(4-Meth­oxy-3-nitro­phen­yl)acetamide

**DOI:** 10.1107/S2414314623002985

**Published:** 2023-04-04

**Authors:** James E. Hines III, Ogad A. Agu, Curtistine J. Deere, Frank R. Fronczek, Rao M. Uppu

**Affiliations:** a Department of Environmental Toxicology, Southern University and A&M College, Baton Rouge, LA 70813, USA; b Department of Chemistry, Louisiana State University, Baton Rouge, LA 70803, USA; University of Aberdeen, United Kingdom

**Keywords:** *N*-alk­oxy­acetanilides, phenacetin congeners, nitro products, crystal structure, hydrogen bonding

## Abstract

The title compound, C_9_H_10_N_2_O_4_, crystallizes with a disordered nitro group in twinned crystals. Both the meth­oxy group and the acetamide groups are nearly coplanar with the phenyl ring, and the C—N—C—O torsion angle [0.2 (4)°] is also insignificantly different from zero. Overall, the 12-atom meth­oxy­phenyl­acetamide group is nearly planar, with an r.m.s. deviation of 0.042 Å. The nitro group is twisted out of this plane by about 30°, disordered into two orientations with opposite senses of twist. In the crystal, the N—H group donates a hydrogen bond to a nitro oxygen atom, generating chains propagating in the [101] direction. The amide carbonyl oxygen atom is not involved in the hydrogen bonding.

## Abstract 2

The title compound crystallizes with a disordered nitro group in twinned crystals. In the crystal, the N—H group donates a hydrogen bond to a nitro oxygen atom, generating chains propagating in the [101] direction. The amide carbonyl oxygen atom is not involved in the hydrogen bonding.

## Graphical abstract

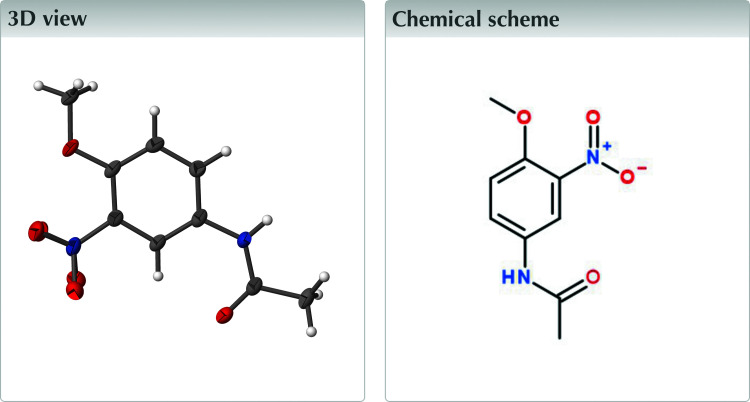

## Structure description

Belonging to the class of 4-alk­oxy­acetanilides (4-AAs), phenacetin [*N*-(4-eth­oxy­phen­yl)acetamide] was the first synthetic fever reducer and non-opioid analgesic to go on the market worldwide as early as the 1890s. It is generally believed that the analgesic effects of 4-AAs are due to their actions on the sensory tracts of the spinal cord, while the anti­pyretic actions arise from their actions on the brain where the temperature set point is lowered (Dalmann *et al.*, 2015 ▸; Flower & Vane, 1972 ▸). *In vivo*, 4-AAs mostly undergo oxidative *O*-de­alkyl­ation to give *N*-(4-hydroxphen­yl)acetamide (Brodie & Axelrod, 1948 ▸; Kapetanović & Mieyal, 1979 ▸), the clinically relevant analgesic, while small portions may undergo de­acyl­ation, producing carcinogenic, kidney-damaging 4-alk­oxy­anilines and/or their N-oxidation products, namely, *N*-(4-alk­oxy­phen­yl)hydroxyl­amine and 1-alk­oxy-4-nitroso­benzene (Prescott, 1980 ▸).

There has been extensive information on phase I and phase II biotransformation of 4-AAs (Estus & Mieyal, 1983 ▸; Hinson, 1983 ▸; Kapetanović & Mieyal, 1979 ▸; Taxak *et al.*, 2013 ▸), but little is known about their biotransformation by non-enzymatic mechanisms, including those mediated by nitric oxide-derived free radical and non-free radical oxidants (*viz*., nitro­gen dioxide, carbonate radical, and per­oxy­nitrous acid). Studies from our laboratory have shown, for instance, that *N*-(4-hy­droxy­phen­yl)acetamide forms nitrated products along with varying amounts of dimers when reacted with the said nitric oxide-derived oxidants under physiologically relevant conditions (Deere *et al.*, 2023 ▸; Uppu & Martin, 2004 ▸). We reason that similar products (or their positional isomers) may be formed in the reactions of 4-AAs with nitric oxide-derived oxidants or other cellular oxidants like the hypochlorite/hypo­chlorous acid conjugate acid/base system (pH ≃ 7.53).

Towards better understanding of these possibilities and to shed light on mol­ecular targets, we have synthesized the title compound, C_9_H_10_N_2_O_4_ [*N*-(4-meth­oxy-3-nitro­phen­yl)acetamide]: crystals grown in water were analyzed by X-ray diffraction. Combined with the recent revelations of mechanisms of action of *N*-(4-hy­droxy­phen­yl)acetamide through indirect activation of CB1 receptors by 4-amino­phenol [hydrolysis product of *N*-(4-hy­droxy­phen­yl)acetamide] and endocannabinoid reuptake inhibitor AM404 (Bertolini *et al.*, 2006 ▸; Zygmunt *et al.*, 2000 ▸), the information presented here may provide useful insights into mol­ecular targets for 4-AAs and their nitrated metabolites.

**Figure 1:**
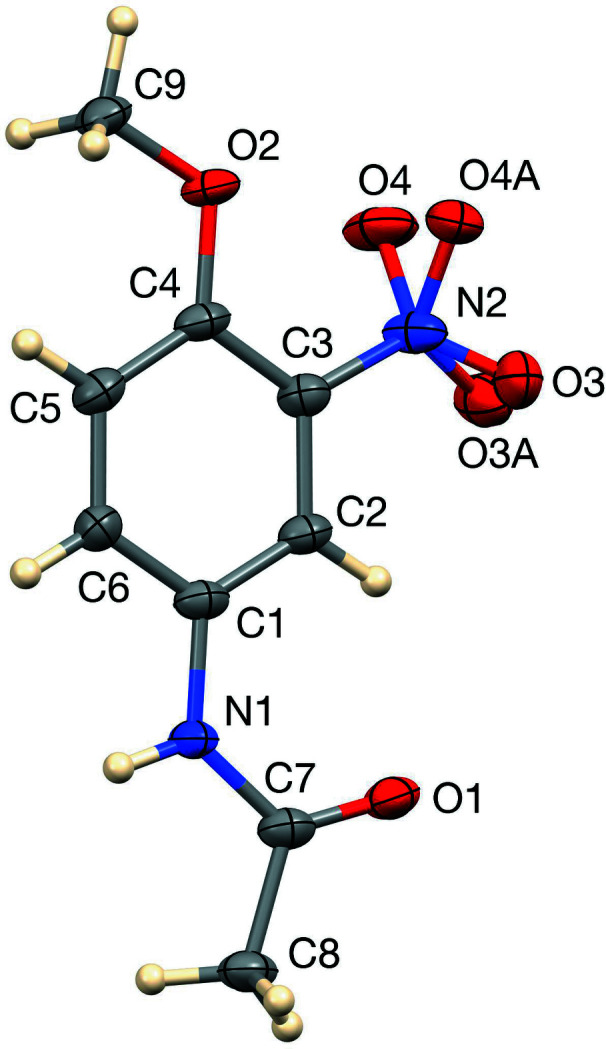
The title mol­ecule with displacement ellipsoids drawn at the 50% probability level.

**Figure 2:**
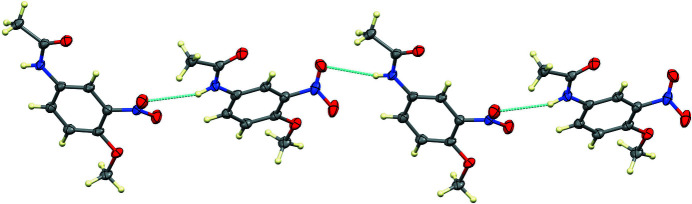
The hydrogen-bonding scheme. Only one orientation of the disordered NO_2_ group is shown.

**Figure 3:**
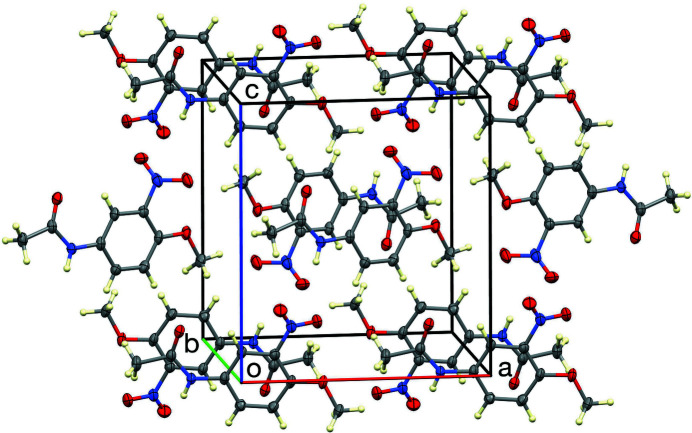
The unit-cell packing. Only one orientation of the disordered NO_2_ group is shown.

The title compound, shown in Fig. 1 ▸, crystallizes with a disordered nitro group in twinned crystals. Both the meth­oxy group and the acetamide groups are nearly coplanar with the phenyl ring, with respective torsion angles 0.0 (4)° for C9—O2—C4—C5 and 4.9 (4)° for C7—N1—C1—C2. The C1—N1—C7—O1 torsion angle is also insignificantly different from zero, 0.2 (4)°. Overall, the atoms of the 12-atom meth­oxy­phenyl­acetamide group are almost coplanar with an r.m.s. deviation of 0.042 Å. The nitro group is twisted out of this plane by 23.5 (2) and 35.6 (2)°, disordered into two orientations with opposite senses of twist. The dihedral angle between the two disordered C—NO_2_ planes is 59.2 (2)°. The N—H group donates inter­molecular hydrogen bonds to the nitro oxygen atom at *x* − 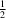 , 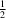 − *y*, *z* − 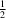 , with an N1⋯O3*A* distance of 3.122 (4) Å (Table 1 ▸), thereby forming chains propagating in the [101] direction, as shown in Fig. 2 ▸. The unit cell is shown in Fig. 3 ▸. Inter­estingly, the amide carbonyl oxygen atom is not involved in the hydrogen bonding.

## Synthesis and crystallization

*N*-(4-Meth­oxy-3-nitro­phen­yl)acetamide was synthesized by the acetyl­ation of 4-meth­oxy-3-nitro­aniline using acetic anhydride. Typically, 20 mmol (3.36 g) of 4-meth­oxy-3-nitro­aniline in 30 ml of glacial acetic acid was refluxed for 2 h with 20% molar excess (24 mmol; 2.46 g) of acetic anhydride. The reaction mixture was stirred continuously during the reaction. In the end, the mixture was dried under vacuum, and the residue was purified by recrystallization twice from deionized water. Single crystals of the title compound were grown from an aqueous solution by slow cooling of a hot and nearly saturated solution.

## Refinement

Crystal data, data collection and structure refinement details are summarized in Table 2 ▸. The crystal chosen for data collection was found to be a three-component nonmerohedral twin with approximate fractions of 0.962: 0.024: 0.014. Refinement was against a twin4.hkl file prepared by TWINABS and the twin fractions were not refined.

## Supplementary Material

Crystal structure: contains datablock(s) I. DOI: 10.1107/S2414314623002985/hb4429sup1.cif

Structure factors: contains datablock(s) I. DOI: 10.1107/S2414314623002985/hb4429Isup2.hkl

Supporting information file. DOI: 10.1107/S2414314623002985/hb4429Isup3.cml

CCDC reference: 2253001

Additional supporting information:  crystallographic information; 3D view; checkCIF report

## Figures and Tables

**Table 1:** Hydrogen-bond geometry (Å, °)

*D*—H⋯*A*	*D*—H	H⋯*A*	*D*⋯*A*	*D*—H⋯*A*
N1—H1*N*⋯O3^i^	0.87 (3)	2.59 (3)	3.410 (5)	157 (2)
N1—H1*N*⋯O3*A* ^i^	0.87 (3)	2.37 (3)	3.122 (4)	145 (3)

Symmetry code: (i) 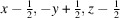 .

**Table 2:** Experimental details

Crystal data	
Chemical formula	C_9_H_10_N_2_O_4_
*M*_r_	210.19
Crystal system, space group	Monoclinic, *P*2_1_/*n*
Temperature (K)	90
*a*, *b*, *c* (Å)	10.8740 (8), 7.0136 (6), 12.2891 (12)
β (°)	92.313 (5)
*V* (Å^3^)	936.48 (14)
*Z*	4
Radiation type	Cu *K*α
μ (mm^−1^)	1.02
Crystal size (mm)	0.32 × 0.09 × 0.04

Data collection	
Diffractometer	Bruker Kappa APEXII DUO CCD
Absorption correction	Multi-scan (*TWINABS*; Bruker, 2016 ▸)
*T*_min_, *T*_max_	0.742, 0.961
No. of measured, independent and observed [*I* > 2σ(*I*)] reflections	2937, 1675, 1360
*R*_int_	0.047
(sin θ/λ)_max_ (Å^−1^)	0.607

Refinement	
*R*[*F* ^2^ > 2σ(*F* ^2^)], *wR*(*F* ^2^), *S*	0.059, 0.176, 1.08
No. of reflections	1675
No. of parameters	160
H-atom treatment	H atoms treated by a mixture of independent and constrained refinement
Δρ_max_, Δρ_min_ (e Å^−3^)	0.28, −0.35

Computer programs: *APEX2* and *SAINT* (Bruker, 2016 ▸), *SHELXT2014/5* (Sheldrick, 2008 ▸), *SHELXL2017/1* (Sheldrick, 2015 ▸), *Mercury* (Macrae *et al.*, 2020 ▸) and *publCIF* (Westrip, 2010 ▸).

## full crystallographic data

### Crystal data

**Table:**

C_9_H_10_N_2_O_4_	*F*(000) = 440
*M**_r_* = 210.19	*D*_x_ = 1.491 Mg m^−^^3^
Monoclinic, *P*2_1_/*n*	Cu *K*α radiation, λ = 1.54184 Å
*a* = 10.8740 (8) Å	Cell parameters from 3893 reflections
*b* = 7.0136 (6) Å	θ = 5.3–69.2°
*c* = 12.2891 (12) Å	µ = 1.02 mm^−^^1^
β = 92.313 (5)°	*T* = 90 K
*V* = 936.48 (14) Å^3^	Needle, yellow
*Z* = 4	0.32 × 0.09 × 0.04 mm

### Data collection

**Table:**

Bruker Kappa APEXII DUO CCD diffractometer	1675 independent reflections
Radiation source: IµS microfocus	1360 reflections with *I* > 2σ(*I*)
QUAZAR multilayer optics monochromator	*R*_int_ = 0.047
φ and ω scans	θ_max_ = 69.4°, θ_min_ = 5.3°
Absorption correction: multi-scan (*TWINABS*; Bruker, 2016)	*h* = −13→13
*T*_min_ = 0.742, *T*_max_ = 0.961	*k* = −8→8
2937 measured reflections	*l* = −14→14

### Refinement

**Table:**

Refinement on *F*^2^	0 restraints
Least-squares matrix: full	Hydrogen site location: mixed
*R*[*F*^2^ > 2σ(*F*^2^)] = 0.059	H atoms treated by a mixture of independent and constrained refinement
*wR*(*F*^2^) = 0.176	*w* = 1/[σ^2^(*F*_o_^2^) + (0.0926*P*)^2^ + 0.7816*P*] where *P* = (*F*_o_^2^ + 2*F*_c_^2^)/3
*S* = 1.08	(Δ/σ)_max_ < 0.001
1675 reflections	Δρ_max_ = 0.28 e Å^−^^3^
160 parameters	Δρ_min_ = −0.35 e Å^−^^3^

### Special details

**Table:**

Geometry. All esds (except the esd in the dihedral angle between two l.s. planes) are estimated using the full covariance matrix. The cell esds are taken into account individually in the estimation of esds in distances, angles and torsion angles; correlations between esds in cell parameters are only used when they are defined by crystal symmetry. An approximate (isotropic) treatment of cell esds is used for estimating esds involving l.s. planes.
Refinement. Twinned crystal. Refinement was vs. an HKLF4 file prepared by TWINABS. All H atoms were located in difference maps and those on C were thereafter treated as riding in geometrically idealized positions with C—H distances of 0.95 Å for phenyl and 0.98 Å for methyl. The coordinates of the amide H atom were refined. *U*_iso_(H) values were constrained to be 1.2*U*_eq_ for the attached atom (1.5 for methyl).

### Fractional atomic coordinates and isotropic or equivalent isotropic displacement parameters (Å^2^)

**Table:**

	*x*	*y*	*z*	*U*_iso_*/*U*_eq_	Occ. (<1)
O1	0.29551 (15)	0.2148 (3)	0.64386 (16)	0.0309 (5)	
O2	0.84160 (14)	0.4030 (3)	0.47776 (15)	0.0291 (5)	
O3	0.6667 (4)	0.4337 (10)	0.7443 (4)	0.0382 (13)	0.500 (6)
O4	0.8325 (3)	0.3096 (8)	0.6813 (4)	0.0450 (16)	0.500 (6)
O3A	0.6722 (4)	0.3056 (10)	0.7606 (3)	0.0377 (13)	0.500 (6)
O4A	0.8073 (3)	0.4813 (6)	0.6859 (3)	0.0366 (13)	0.500 (6)
N1	0.34765 (17)	0.1800 (3)	0.46794 (18)	0.0246 (5)	
H1N	0.323 (3)	0.145 (4)	0.403 (3)	0.030*	
N2	0.72034 (18)	0.3692 (3)	0.67532 (19)	0.0314 (6)	
C1	0.4726 (2)	0.2346 (3)	0.4747 (2)	0.0248 (6)	
C2	0.5355 (2)	0.2771 (3)	0.5717 (2)	0.0248 (6)	
H2	0.495195	0.269873	0.638738	0.030*	
C3	0.6593 (2)	0.3309 (4)	0.5696 (2)	0.0249 (6)	
C4	0.7224 (2)	0.3447 (3)	0.4729 (2)	0.0252 (6)	
C5	0.6570 (2)	0.2991 (4)	0.3768 (2)	0.0278 (6)	
H5	0.696789	0.305718	0.309488	0.033*	
C6	0.5349 (2)	0.2441 (3)	0.3779 (2)	0.0255 (6)	
H6	0.492590	0.212081	0.311263	0.031*	
C7	0.2676 (2)	0.1729 (3)	0.5507 (2)	0.0260 (6)	
C8	0.1392 (2)	0.1083 (4)	0.5162 (2)	0.0316 (6)	
H8A	0.102811	0.039286	0.576348	0.047*	
H8B	0.143220	0.024068	0.452865	0.047*	
H8C	0.088432	0.219750	0.497165	0.047*	
C9	0.9015 (2)	0.4168 (4)	0.3751 (2)	0.0308 (6)	
H9A	0.906509	0.289913	0.342208	0.046*	
H9B	0.984699	0.468291	0.387608	0.046*	
H9C	0.854022	0.501614	0.325949	0.046*	

### Atomic displacement parameters (Å^2^)

**Table:**

	*U*^11^	*U*^22^	*U*^33^	*U*^12^	*U*^13^	*U*^23^
O1	0.0196 (9)	0.0424 (11)	0.0312 (12)	−0.0022 (7)	0.0055 (7)	0.0013 (8)
O2	0.0149 (8)	0.0369 (11)	0.0359 (11)	−0.0033 (6)	0.0060 (7)	0.0021 (8)
O3	0.036 (2)	0.052 (4)	0.027 (2)	−0.003 (2)	0.0027 (17)	−0.009 (2)
O4	0.0188 (19)	0.078 (4)	0.037 (3)	−0.0051 (18)	−0.0031 (15)	0.010 (2)
O3A	0.033 (2)	0.052 (4)	0.027 (2)	−0.0082 (19)	−0.0026 (16)	0.002 (2)
O4A	0.0236 (19)	0.048 (3)	0.039 (2)	−0.0111 (16)	0.0014 (15)	−0.0081 (18)
N1	0.0163 (10)	0.0324 (12)	0.0251 (12)	−0.0046 (7)	0.0012 (8)	−0.0011 (9)
N2	0.0188 (10)	0.0402 (13)	0.0349 (14)	−0.0045 (9)	−0.0023 (9)	0.0080 (11)
C1	0.0164 (11)	0.0231 (12)	0.0348 (15)	0.0006 (8)	0.0023 (9)	0.0026 (10)
C2	0.0172 (11)	0.0254 (13)	0.0320 (15)	−0.0010 (8)	0.0048 (9)	0.0030 (10)
C3	0.0196 (12)	0.0275 (13)	0.0276 (14)	0.0002 (9)	0.0015 (9)	0.0024 (10)
C4	0.0156 (11)	0.0237 (12)	0.0368 (16)	−0.0005 (8)	0.0061 (9)	0.0038 (10)
C5	0.0229 (12)	0.0311 (14)	0.0299 (15)	0.0003 (9)	0.0081 (10)	0.0019 (11)
C6	0.0223 (12)	0.0283 (13)	0.0261 (14)	0.0009 (9)	0.0029 (9)	−0.0028 (10)
C7	0.0177 (12)	0.0259 (13)	0.0346 (16)	0.0002 (9)	0.0019 (10)	0.0038 (10)
C8	0.0177 (12)	0.0337 (14)	0.0433 (17)	−0.0034 (9)	0.0016 (10)	−0.0016 (12)
C9	0.0192 (12)	0.0397 (15)	0.0343 (15)	−0.0022 (10)	0.0087 (10)	0.0011 (11)

### Geometric parameters (Å, º)

**Table:**

O1—C7	1.209 (3)	C2—H2	0.9500
O2—C4	1.358 (3)	C3—C4	1.399 (3)
O2—C9	1.446 (3)	C4—C5	1.391 (4)
O3—N2	1.142 (5)	C5—C6	1.383 (3)
O4—N2	1.288 (5)	C5—H5	0.9500
O3A—N2	1.271 (5)	C6—H6	0.9500
O4A—N2	1.233 (4)	C7—C8	1.511 (3)
N1—C7	1.366 (3)	C8—H8A	0.9800
N1—C1	1.411 (3)	C8—H8B	0.9800
N1—H1N	0.87 (3)	C8—H8C	0.9800
N2—C3	1.459 (3)	C9—H9A	0.9800
C1—C2	1.383 (4)	C9—H9B	0.9800
C1—C6	1.394 (3)	C9—H9C	0.9800
C2—C3	1.400 (3)		
			
C4—O2—C9	116.4 (2)	C6—C5—C4	120.9 (2)
C7—N1—C1	127.4 (2)	C6—C5—H5	119.5
C7—N1—H1N	119.2 (19)	C4—C5—H5	119.5
C1—N1—H1N	113.4 (19)	C5—C6—C1	121.4 (2)
O4A—N2—O3A	118.5 (3)	C5—C6—H6	119.3
O3—N2—O4	126.7 (4)	C1—C6—H6	119.3
O3—N2—C3	120.4 (3)	O1—C7—N1	123.6 (2)
O4A—N2—C3	122.0 (3)	O1—C7—C8	122.2 (2)
O3A—N2—C3	118.8 (3)	N1—C7—C8	114.3 (2)
O4—N2—C3	112.7 (3)	C7—C8—H8A	109.5
C2—C1—C6	119.0 (2)	C7—C8—H8B	109.5
C2—C1—N1	123.4 (2)	H8A—C8—H8B	109.5
C6—C1—N1	117.6 (2)	C7—C8—H8C	109.5
C1—C2—C3	119.0 (2)	H8A—C8—H8C	109.5
C1—C2—H2	120.5	H8B—C8—H8C	109.5
C3—C2—H2	120.5	O2—C9—H9A	109.5
C4—C3—C2	122.6 (2)	O2—C9—H9B	109.5
C4—C3—N2	121.5 (2)	H9A—C9—H9B	109.5
C2—C3—N2	115.9 (2)	O2—C9—H9C	109.5
O2—C4—C5	124.1 (2)	H9A—C9—H9C	109.5
O2—C4—C3	118.9 (2)	H9B—C9—H9C	109.5
C5—C4—C3	117.0 (2)		
			
C7—N1—C1—C2	4.9 (4)	C9—O2—C4—C5	0.0 (4)
C7—N1—C1—C6	−175.6 (2)	C9—O2—C4—C3	−179.3 (2)
C6—C1—C2—C3	0.9 (4)	C2—C3—C4—O2	178.2 (2)
N1—C1—C2—C3	−179.6 (2)	N2—C3—C4—O2	−2.8 (4)
C1—C2—C3—C4	0.4 (4)	C2—C3—C4—C5	−1.1 (4)
C1—C2—C3—N2	−178.6 (2)	N2—C3—C4—C5	177.9 (2)
O3—N2—C3—C4	147.7 (4)	O2—C4—C5—C6	−178.8 (2)
O4A—N2—C3—C4	29.0 (4)	C3—C4—C5—C6	0.5 (4)
O3A—N2—C3—C4	−160.7 (4)	C4—C5—C6—C1	0.8 (4)
O4—N2—C3—C4	−37.3 (4)	C2—C1—C6—C5	−1.5 (4)
O3—N2—C3—C2	−33.3 (5)	N1—C1—C6—C5	179.0 (2)
O4A—N2—C3—C2	−152.0 (3)	C1—N1—C7—O1	0.2 (4)
O3A—N2—C3—C2	18.4 (5)	C1—N1—C7—C8	179.9 (2)
O4—N2—C3—C2	141.8 (3)		

### Hydrogen-bond geometry (Å, º)

**Table:**

*D*—H···*A*	*D*—H	H···*A*	*D*···*A*	*D*—H···*A*
N1—H1*N*···O3^i^	0.87 (3)	2.59 (3)	3.410 (5)	157 (2)
N1—H1*N*···O3*A*^i^	0.87 (3)	2.37 (3)	3.122 (4)	145 (3)

Symmetry code: (i) *x*−1/2, −*y*+1/2, *z*−1/2.
